# *Bdellovibrio* and Like Organisms in Lake Geneva: An Unseen Elephant in the Room?

**DOI:** 10.3389/fmicb.2020.00098

**Published:** 2020-02-14

**Authors:** Jade A. Ezzedine, Louis Jacas, Yves Desdevises, Stéphan Jacquet

**Affiliations:** ^1^Université Savoie Mont-Blanc, INRAE, CARRTEL, Thonon-les-Bains, France; ^2^CNRS, Biologie Intégrative des Organismes Marins, Observatoire Océanologique, Sorbonne Université, Banyuls-sur-Mer, France

**Keywords:** *Bdellovibrio* and like organisms, myxobacteria, predatory bacteria, microbial loop, Lake Geneva, metabarcoding

## Abstract

When considering microbial biotic interactions, viruses as well as eukaryotic grazers are known to be important components of aquatic microbial food webs. It might be the same for bacterivorous bacteria but these groups have been comparatively less studied. This is typically the case of the *Bdellovibrio* and like organisms (BALOs), which are obligate bacterial predators of other bacteria. Recently, the abundance and distribution of three families of this functional group were investigated in perialpine lakes, revealing their presence and quantitative importance. Here, a more in-depth analysis is provided for Lake Geneva regarding the diversity of these bacterial predators at different seasons, sites and depths. We reveal a seasonal and spatial (vertical) pattern for BALOs. They were also found to be relatively diverse (especially *Bdellovibrionaceae*) and assigned to both known and unknown phylogenetic clusters. At last we found that most BALOs were positively correlated to other bacterial groups, mainly Gram-negative, in particular *Myxococcales* (among which many are predators of other microbes). This study is the first shedding light on this potentially important bacterial killing group in a large and deep lake.

## Introduction

In aquatic ecosystems, it is now well-known that microbes play many important roles. Viruses, archaea, bacteria, phytoplankton, and all other unicellular eukaryotes have been identified as key actors for biomass production, matter and nutrient (re)cycling, biotic interactions (including predation and symbioses such as parasitism) and energy transfer throughout the food webs (Kirchman et al., 1982; Azam et al., 1983; Fuhrman and Noble, 1995; Fenchel, 2008). On one hand, for instance, marine bacteria consume 20 to 60% of the organic carbon from primary production (Azam and Malfatti, 2007). Through microbial respiration, the produced dissolved organic matter returns to the atmosphere as CO_2_ that will, in turn, serve the photosynthesis process of auto- or mixotrophic organisms (Bolan et al., 2011). On the other hand, DOM consumed by bacteria is transferred to higher trophic levels, i.e. small flagellates and ciliate protozoans. This microbial loop, to which we can add the lytic action of (bacterio)phages, explains why, in general, the abundance of bacteria remains relatively stable from year to year, whatever the ecosystem studied (Jacquet et al., 2010). If bacteriophage-induced mortality rate and protozoan grazing have been reported as the main biotic pressure on bacterial communities (Fuhrman and Noble, 1995; Pernthaler, 2005), with many consequences on bacterial abundance and composition, transfer of carbon and nutrients to higher trophic levels and, *in fine*, on the whole ecosystem functioning (Motegi et al., 2009; Ram et al., 2014), other biotic interactions such as bacteria predating other bacteria have comparatively received lower attention.

Indeed, among the biological compartments that have been largely neglected, there are the predatory bacteria that depend on other bacteria for their nutrient requirements, growth and survival. Clearly, less is known about these predators present in many bacterial classes (Jurkevitch and Davidov, 2006) and which have been proposed as a potential important evolutionary driving force (Erken et al., 2013; Pérez et al., 2016). The bacterial predators can be numerous and diverse in different aspects. Some are facultative hunters, i.e. their hunting behavior is only employed when nutrients become scarce, while others are obligate predators, hunting either in packs or alone. Among these predators, some bacteria are unique obligate predators, the *Bdellovibrio* and like organisms (BALOs).

*Bdellovibrio* and like organisms have certainly been the most studied group of obligate predatory bacteria (Jurkevitch and Davidov, 2006). They are Gram-negative bacteria that have the same hunting mode, characterized by two life cycle strategies: periplasmic life cycle (inside consumption of the prey bacterium) vs. epibiotic (external consumption). This group encompasses few genera, 6 so far, yet their classification has changed many times over the last two decades. Today, these bacteria are classified in 5 families and 1 order. Originally, the *Bdellovibrionaceae*, *Peredibacteraceae*, *Bacteriovoracaceae*, *Halobacteriovoraceae* and *Pseudobacteriovoracaceae*, belonged to the Deltaproteobacteria. Recently, they moved to the Oligoflexia class (Hahn et al., 2017). Only one genus, referred to as *Micavibrio*, is classified differently and belongs to Alphaproteobacteria. Among these obligate predators, *Bdellovibrio bacteriovorus* is by far the most studied BALOs. *B. bacteriovorus* life cycle was described *via* microscopic observation and from a genomic perspective (Rendulic et al., 2004).

*Bdellovibrio* and like organisms are very similar in shape and size, i.e. a comma shape with a flagellum, measuring between 0.2 and 0.5 μm in width and 0.5 to 2.5 μm in length (Crossman et al., 2013). They grow and reproduce on other Gram-negative bacteria. BALOs are ubiquitous in a multitude of habitats such as soils, salt, and fresh waters (Jurkevitch, 2012). Therefore, they have been reported as possible important bioagents controlling bacterial populations. As a result, BALOs are medically considered as antibiotic replacement giving their hunting behavior toward Gram-negative pathogens (Willis et al., 2016) and indirectly through their reservoir of enzyme-based antimicrobial substances (Rendulic et al., 2004). In addition, BALOs like bacteriophage and protozoans are likely to impact the structure and dynamics of some bacterial populations or communities (Davidov and Jurkevitch, 2004; Williams et al., 2016) but studies highlighting this are still missing.

Unlike soil and saltwater, fresh waters especially lakes have been less examined (Paix et al., 2019). It is however hypothesized that these predatory bacteria play, at some extent, important roles in the structure and functioning of lacustrine microbial communities (Chen and Williams, 2012). Using data obtained during the TRANSLEM project, a study of the diversity of bacteria and archaea in Lake Geneva with high-throughput sequencing of the 16S rRNA gene, we focused on BALOs in the present work. Our study sheds light on the diversity and distribution of these predators, as well as their relationships with the bacterial community and environmental factors in the largest natural deep lake of Western Europe. We show that these bacteria are phylogenetically diverse in Lake Geneva, suggesting that they can be important players in the (aquatic microbial) game and thus should deserve particular attention.

## Materials and Methods

### Study Site

Lake Geneva is a deep and large warm monomictic lake (surface area: 580 km^2^; volume: 89 km^3^; maximum depth: 309.7 m; mean depth: 152.7 m), located in the western part of the Alps at an altitude of 372 m. Lake Geneva has been monitored since 1974 as a part of a long-term water quality and biological monitoring program. Sampling has been continuously undertaken in the middle of the lake at the deepest point, referred to as SHL2, once or twice a month (Supplementary Figure 1). This scientific survey revealed the lake has switched from an oligotrophic to eutrophic state with annual phosphorus concentrations reaching 90 μgP L^–1^ in 1979 (Anneville et al., 2002). Thanks to effective management measures, Lake Geneva turned back to a mesotrophic state in the early 2000s with total phosphorus concentrations about 20 μg L^–1^ in 2010 (Jacquet et al., 2014).

### Sampling Strategy

During the TRANSLEM project, we collected water samples at three sites, including the reference station SHL2. These sites referred to as pt2, pt4 (SHL2) and pt6 (Supplementary Figure 1), separated from each other by approximately 14 km, were sampled at four different dates and seasons: February 20 (TL1), June 4 (TL2), August 7 (TL3) and November 20 (TL4) 2014, and at three different depths, i.e. 2, 15, and 200 m. It is noteworthy that pt2 could not be sampled on June 4 due to bad weather conditions. A filtration system was installed on the boat and a volume of 300 mL of each water sample was consecutively filtered through 5, then 2 and finally 0.2 μm polycarbonate filters, 47 mm in diameter (Milipore) to obtain three different size fractionations. Filters were then kept at −20°C until DNA extraction.

Descriptors such as temperature, pH, conductivity, chlorophyll *a*, and dissolved oxygen concentrations of the water column were measured using a multiparameter probe (Sea & Sun technology GMBH). Transparency was measured using a normalized 25 cm diameter Secchi disk. Nutrients such as total organic carbon (TOC) and nutrient concentrations, i.e. total nitrogen (TN), dissolved ammonium (NH_4_-N), dissolved nitrates (NO3-N), total phosphorus (TP), and orthophosphates (PO_4_-P) were measured only at SHL2 at the different depths and dates, according to the standard French protocols AFNOR.

### DNA Extraction

The filters were subjected to DNA extraction using a homemade protocol with GenEluteTM-LPA (Sigma-Aldrich) solution. The protocol started with a lysis step in Eppendorf tubes by adding 300 μL of TE buffer (TRIS 1M – pH 8, EDTA 0.5M – pH 8) and 200 μL of a lysis solution (TRIS 1M – pH 8, EDTA 0.5M – pH 8 and sucrose 0.7M). Then, a thermic shock was carried out by placing the tubes at −80°C for 15 min and thawed into a block heater at 55°C for 2 min. Next, 50 μL of a 10% sodium dodecyl sulfate (SDS) and 10 μL of proteinase K (20 mg mL^–1^) were added to the solution. The solution was incubated at 37°C for 1 h with gentle stirring and placed again in the block heater at 55°C for 20 min. After a quick centrifugation step (13,000 rpm at 4°C for 3 min), the supernatant was collected. Then, 50 μL of sodium acetate (3M – pH 5.2) and 1 μL of GenEluteTM-LPA (Sigma-Aldrich, 25 μg μL^–1^) were added. One volume of isopropanol was then added and the tubes were centrifuged for 10 min at 12,000*g* and 4°C. The supernatant was discarded and 2 washing rounds using ethanol (80%) were carried to purify the DNA. The remaining ethanol was evaporated using a Speed-Vac for 20 min. Finally, 30 μL of TE were added and tubes were left for 1 h at 37°C. DNA concentration was measured using a NanoDrop 1000 spectrophotometer. Afterward, all tubes were stored at −20°C until analysis.

### PCR and Sequencing

DNA extracts were set at 25 ng μL^–1^. DNA extracted from the 5 and 2 μm filters were pooled to avoid material loss and used to estimate the attached fraction of the bacteria. Comparatively, the 0.2 < fraction <2 μm corresponded to the free-living bacteria. The PCR amplification of 16S rRNA gene fragments was performed using a tagged forward primer 515F (GTGYCAGCMGCCGCGGTA) (Wang and Qian, 2009) and a tagged reverse primer 909R (CCCCCGYCAATTCMTTTRAGT) (Wang et al., 2018). The number of samples was 66 and a PCR replicate was made for each sample giving a total of 132 samples. Each sample was identified with a different tag. PCR mixture volume was 30 μL and consisted of (final concentration): 1x buffer, 0.5 mM dNTP, 1.5 mM MgCl_2_, 0.5 mg mL^–1^ bovine serum albumin (BSA) and 0.75 U Biotaq DNA polymerase (Bioline). In a second step, a unique combination of tagged primers (forward and reverse) was added to each sample. Finally, 1 μL of template DNA (25 ng μL^–1^) was added. A negative control was included and the PCR program was as follows: 95°C – 2 min, 30 × (94°C – 30 s, 58°C – 30 s, 72°C – 30 s), with a final extension step at 72°C for 5 min. Agarose gel analysis was performed for verification of the PCR products. When samples showed a non-specific band (approximately 550 bp) close to the target band (approximately 450 bp), the target bands were then captured using Pippin prep system (sage science) following the manufacturer’s instructions. Then these captured DNA were checked with TapeStation (Agilent 2200) system for size and quality assessment following the manufacturer’s instructions. All amplified DNA were quantified using the Quant-iT PicoGreen ds DNA Reagent kit (Invitrogen) and fluorescence was read using the plate reader Fluoroskan Ascent FL. All DNA samples were then pooled as one equimolar tube. DNA were then purified using the Clean PCR kit (CleanNA) according to the manufacturer’s instructions to remove dNTP and dimers. Again, the pool was quantified using PicoGreen. Then the pool tube containing the 132 different tagged DNA was sent to the GATC-Eurofins platform for DNA sequencing using Illumina Hiseq 300 paired end technology to get 2 × 150 bp amplicons.

### Bioinformatics Pipeline

Two files of raw data (5′–3′ and 3′–5′) in fastq format were received from the sequencing platform. Files were processed using the pipeline developed by Frederic Mahé known as “Fred’s metabarcoding pipeline” found at https://github.com/frederic-mahe/swarm/wiki/Fred’s-metabarcoding-pipeline. It combines programs such as Vsearch (Rognes et al., 2016), Cutadapt (Martin, 2011), and Swarm (Mahé et al., 2015). All default parameters of the pipeline were left unchanged except when mentioned. Briefly, the pipeline starts with merging reads using Vsearch. Next, Cutadapt is used to demultiplex the sequences. Here, each sequence is assigned to its sample in an individual file by its tag. Primers are also removed and sequence containing ambiguous bases are discarded. In each file, sequences are dereplicated using Vsearch. Then all files are assembled as one file and dereplicated in the process. The Swarm algorithm was then used to cluster the sequences. In this step, the “d” parameter (i.e. the number of different nucleotides between sequences) was changed from 1 to 13 in order to be close to the identity threshold of 97% which define same species (Schloss and Handelsman, 2005). Next, *de novo* chimera detection was applied to representative sequences of each cluster using Vsearch. The representative sequences were taxonomically assigned to a reference database downloaded from arb-SILVA (release number 132; Quast et al., 2013) and prepared as required. Next, a python script from the pipeline was used to build the final OTU table. The OTU table was then filtered with three different filters. The first one discarded all sequences flagged as chimera. The second filter removed sequences with a quality score lower than 0.0002. The final filter removed OTUs having less than 3 reads in a sample, unless such OTUs were present in 2 or more samples. *In fine*, we considered only OTUs shared among the two PCR replicates.

### Statistical Analysis

Statistical analyses and plots were performed using R, version 3.5.0 (R Core Team, 2018) and ggplot2 (Wickham et al., 2018). The OTU table was transformed to relative abundance using the “decostand” function from the vegan package (Oksanen et al., 2019). Alpha diversity indices (i.e. Shannon and Pielou) were calculated with the OTUtable package (Linz, 2018). Simpson and inverse Simpson were calculated using the vegan package (Oksanen et al., 2019). The indexes comparison for each condition (Site, Month, Depth, and Filter) was performed using the Kruskal–Wallis test. When the *p*-value of Kruskal–Wallis test was inferior to 0.05 (alpha), a Dunn test was performed with Bonferroni correction (Dinno, 2017). The richness plot found in the Supplementary Figure 2 was made using the “rarecurve” function in vegan. An NMDS to illustrate beta diversity was computed using the “metaMDS” function from vegan and also the goeveg package (Goral and Schellenberg, 2017). A Simper test was also performed with vegan to access the contribution of each OTU to the observed dissimilarity between samples. Regarding environmental variables, they were only available for pt4 (SHL2). OTUs found here were extracted and if an OTU had no read in a site, we removed it from the final OTU table. The data was also transformed as log(1 + x) in order to stabilize the OTU dataset. Then, another NMDS with environmental variables and a CCA were performed on that table following the online tutorial of Umer Zeeshan Ijaz (Torondel et al., 2016) found at http://userweb.eng.gla.ac.uk/umer.ijaz/bioinformatics/ecological.html. Co-occurrence network analysis was performed on all sites following Ju Feng’s R and python scripts (Ju et al., 2014; Ju and Zhang, 2015; Hu et al., 2017) found at https://github.com/RichieJu520/Co-occurrence_Network_Analysis. A bacterial co-occurrence network was built with bacterial orders including *Bdellovibrionales* and *Bacteriovoracales*. The network was composed of 92 nodes (bacterial orders) and 461 undirected edges or connections. The bacterial OTU table used for the network was assigned and curated to the order level. Only positive interactions between community members were considered. The Spearman correlation and *p*-value cutoffs were set to 0.6 and 0.01 in the script. As for the C-score of the network, it was performed with the R package EcoSimR (Gotelli et al., 2015) based on a presence/absence OTU matrix. The visualization and customization of the network was done with Gephi software (Bastian et al., 2009).

### Phylogeny

On one hand, 18 BALOs reference sequences (including type species) of the 16S rRNA (6 for the genus *Bdellovibrio*, 5 for *Peredibacter*, 5 for *Bacteriovorax*, and 2 for *Micavibrio*) were downloaded from NCBI (Benson et al., 2013, Supplementary Table 5). On the other hand, the assigned OTU sequences of BALOs we found (31 for the family *Bdellovibrionaceae*, 8 for *Peredibacteraceae*, 6 for *Bacteriovoracaceae*, and 9 for the order *Micavibrionales*) were used to construct the phylogenetic tree. All sequences were aligned using the program MUSCLE alignment (Edgar, 2004) in MEGA7 (Kumar et al., 2016). The ends of all sequences were trimmed at 5′ and 3′, making reference and OTU sequences of equal length. Next, the alignment was improved using Gblocks 0.91b (Castresana, 2000) with “Minimum Length of a block” set to 5. ModelGenerator v.85 (Keane et al., 2006) was used to select the best nucleotide substitution model (GTR + I + G) under corrected Akaike information criterion (AICc) (Akaike, 1973) with 4 discrete gamma categories. The tree was built using the Maximum likelihood method with PhyML 3.1 (Guindon et al., 2010) with 100 bootstrap replicates. A Bayesian tree was also constructed using MrBayes 3.2.7a (Ronquist et al., 2012) program with 500,000 generations and a burn-in value of 25%.

## Results

### Diversity

Each raw data file contained 55,679,272 reads with read length equal to 301 bp. The merge paired-reads process yielded 49,545,789 reads. In this step, 11% of reads were lost, and the median read length was 437 bp. After demultiplexing or sorting each read to its sample and after a first dereplication, the number of reads reached 11,079,438, with a median length of 377 bp. The second dereplication of reads after reuniting them in one file gave 6,861,908 reads. The clustering process yielded 127,052 representative sequences, including 107,120 chimeric sequences and 19,391 non-chimeric sequences. The median read length after the clustering was 376 bp. After applying the aforementioned filter, and after selecting only bacterial taxonomic assignment and curating the ambiguous assignments, the final number of OTUs was 7,987. It is worth mentioning that the median percentage of identity between representative sequences and database (arb-SILVA) sequences for taxonomic assignment was about 97.4%. The lowest and highest identity percentages were 50.3 and 100% respectively, with an average value of 94.8%.

Among (facultative and obligate) predatory bacteria, BALOs constituted the group with the largest number of reads, representing only 0.61% of total bacteria reads. However, the *Myxococcales* order had the largest number of OTUs, i.e. 240 OTUs, representing 3% of total bacterial OTUs. BALOs were in second position with 130 OTUs, representing 1.63% of total bacteria OTUs (Table 1). It is noteworthy here that the detailed analysis of the OTUs revealed the presence of *Halobacteriovorax* (i.e. a marine group) but after a more in-depth inspection of the sequences using NCBI-BLAST, it was clear that these sequences were wrongly assigned. They corresponded to *Bacteriovoracaceae*. *Bdellovibrionaceae* hold the largest number of OTUs, i.e. 70 OTUs. They also had the higher number of reads (0.585%) among the BALOs (Table 2). At last, when considering the OTUs common to the two replicates, *Bdellovibrionaceae* OTUs remained the most represented (Table 3).

**TABLE 1:** Metabarcoding analysis of 16S rRNA gene sequences revealing all the predatory bacteria found in Lake Geneva through numbers and % of their OTUs and reads, obtained at the three sampled sites and depths all seasons confounded.

Taxa	OTU count and percentage	Reads number and percentage
***Bdellovibrio* and like organisms**		
*Bdellovibrionales*	70(0.88%)	174712(0.58538%)
*Bacteriovoracales*	33(0.41%)	5569(0.01866%)
*Micavibrionales*	27(0.34%)	1998(0.00669%)
*Cellulophaga*	1(0.01%)	70(0.00023%)
*Cytophaga*	22(0.28%)	3323(0.01113%)
*Herpetosiphon*	2(0.03%)	35(0.00012%)
*Lysobacter*	1(0.01%)	58(0.00019%)
*Myxococcales*	240(3.00%)	44017(0.14748%)
*Saprospiraceae*	97(1.21%)	83000(0.27809%)
*Stenotrophomonas*	4(0.05%)	12349(0.04138%)
*Vampirovibrionales*	5(0.06%)	74(0.00025%)
**Total predatory bacteria**	502(6.29%)	325205(1.08961%)
**Total for all bacteria**	7987(100%)	29846037(100%)

**TABLE 2:** Numbers and % of BALOs OTUs and reads obtained at the three sampled sites and depths all seasons confounded.

Taxa of *Bdellovibrio* and like organisms	OTU count and percentage	Reads number and percentage
*Bdellovibrionaceae*	70(0.88%)	174712(0.58538%)
*Micavibrionales*	27(0.34%)	1998(0.00669%)
*Bacteriovoracaceae*	18(0.23%)	2089(0.00700%)
*Peredibacteraceae*	15(0.19%)	3480(0.01166%)
**Total *Bdellovibrio* and like organisms**	130(1.63%)	182279(0.61073%)
**Total for all bacteria**	7987(100%)	29846037(100%)

**TABLE 3:** Numbers of OTUs for BALOs only.

*Bdellovibrio* and like organisms	R1 OTU count	R2 OTU count	R1 read count	R2 read count	Number of shared OTU between R1 and R2	Number of reads for shared OTU between R1 and R2
*Bdellovibrionaceae*	59	57	87779	86933	31	173393
*Micavibrionales*	24	24	1044	954	9	1708
*Bacteriovoracaceae*	16	14	1129	960	6	1860
*Peredibacteraceae*	13	12	2020	1460	8	3270
**Total**	112	107	91972	90307	54	180231
						

### Phylogeny

**FIGURE 1:**
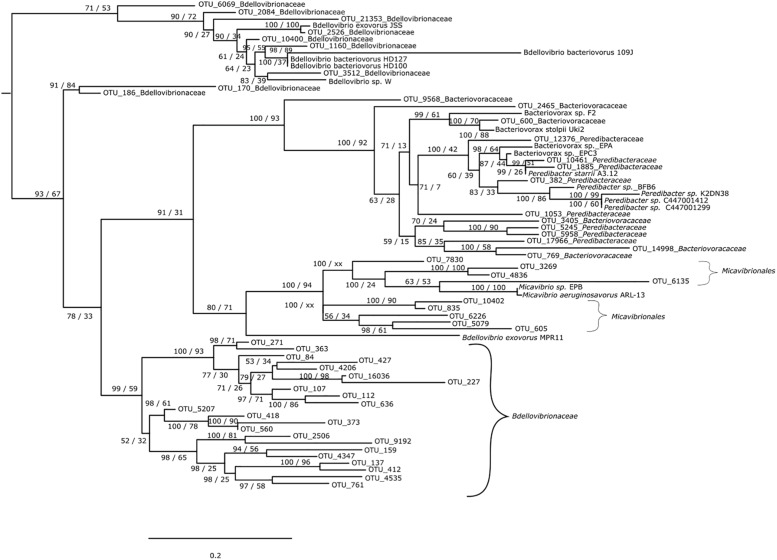
Phylogenetic tree of BALOs environmental sequences with reference database sequence. The tree is based on maximum likelihood analysis and Bayesian inference. Posterior probability (PP) values followed by bootstrap values are added to the left of a node when possible (PP/BS). *Vampirovibrio chlorellavorus* (not shown) was used as an outgroup to root the tree.

For the *Bdellovibrionaceae*, OTU 2526 was closely related to *Bdellovibrio exovorus* JSS while OTU 1160 was closer to *B. bacteriovorus* HD100, HD127 and 109J and OTU 3512 to *Bdellovibrio* sp. W. OTUs 6069, 2084, 21353, 10400 clustered among these individuals (Figure 1). The other OTUs were apart since they did not cluster with any known *Bdellovibrio* sequences and formed two distinct groups (OTUs 170 and 186 vs. OTUs 271, 363, 84, 427, 4206, 16036, 227, 107, 112, 636, 5207, 418, 373, 560, 2506, 9192, 159, 4347, 137, 412, 4535, and 761). For the *Bacteriovoracaceae* two categories were detected. The first group was composed of some *Bacteriovoracaceae* OTUs related to reference sequences of *Bacteriovorax*; it was the case for OTU 600 closely related to *Bacteriovorax stolpii* Uki2 and *Bacteriovorax* sp. F2. The same discrimination was observed for the *Peredibacteraceae:* OTUs 10461 and 1885 were closely related to *Peredibacter starrii* A3.12, OTU 382 to *Peredibacter* sp. BFB6, K2DN38, C114001412 and C114001299. Some OTUs from these families were more difficult to assign: OTU 3405 of *Bacteriovoracaceae* mixed with OTUs 5245 and 5958 of *Peredibacteraceae*, OTU 17966 of *Peredibacteraceae* mixed with OTUs 14998 and 769 of *Bacteriovoracaceae*. At last for the *Micavibrionales* OTUs, they were close to the two reference sequences of *Micavibrio* sp. EPB and *M. aeruginosavorus* ARL-13.

### Distribution and Dynamics

**FIGURE 2:**
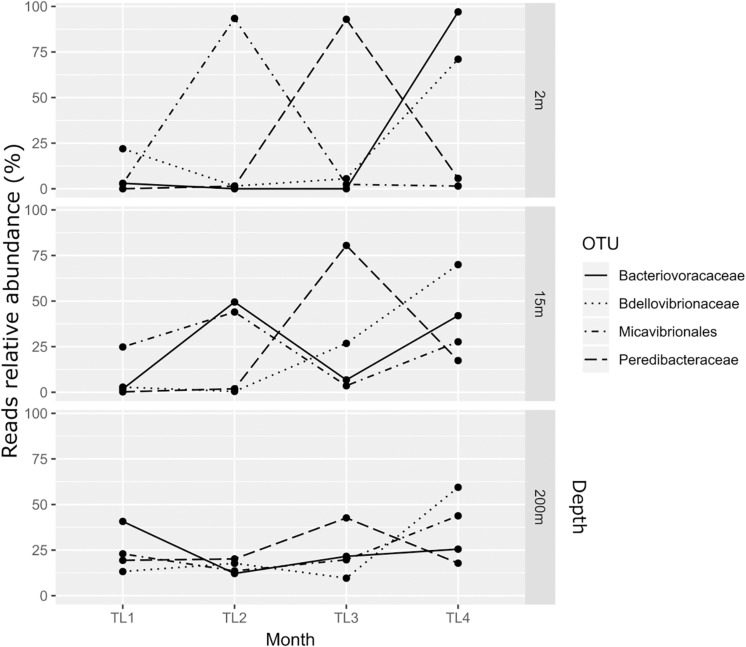
Variation in BALOs relative abundance of reads at the different depths and months.

The relative abundance of BALOs’ reads varied with depth and month (Figure 2). At 2 m the *Bdellovibrionaceae* were abundant in November (TL4) in contrast to the other months with 71% of reads, vs. 22% in February (TL1), 1.5% in June (TL2), and 5.5% in August (TL3). This pattern was also observed at 15 and 200 m in November (TL4), with a proportion of 70 and 59.4% of reads respectively. Similarly, *Bacteriovoracaceae* were also abundant at fall, with 97% of reads at 2 m in November. However, June (TL2) revealed a higher abundance of reads at 15 m representing 49.5% and February (TL1) was richer at 200 m with 40.7%. *Peredibacteraceae* were dominant in summer, with 92.9% of reads in August (TL3) at 2 m, 80.5% at 15 m and 42.6% at 200 m. At last, *Micavibrionales* reads were merely abundant in June (TL2) at 2 m (93.4%) and 15 m (44.0%). At 200 m, November (TL4) held the highest number of reads, 43.8%.

**FIGURE 3:**
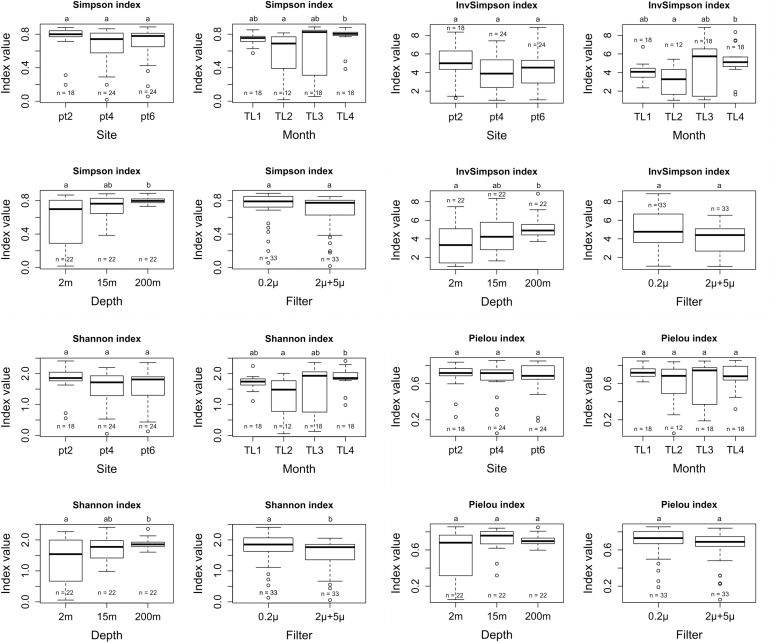
Simpson, InvSimpson, Shannon, and Pielou alpha diversity metrics for site, month, depth, and filter variables.

There was no significant differences for Simpson, Inv-Simpson, Shannon and Pielou indexes between the different sites. A similar result was found when considering the “filter parameter,” i.e. when comparing the 0.2 < filter < 2 μm (free-living cells) vs. < 2 + 5 μm (attached and bigger cells) filters. An exception was observed for the Shannon index (*p*-value = 0.03489), with a higher value for the 0.2 μm filter (Figure 3). Significant differences were observed for Simpson (Inv-Simpson) (*p*-value = 0.009275) and Shannon (*p-*value = 0.01315) indexes. The difference was between June (TL2) and November (TL4) with higher values for the November month according to the Dunn test (Simpson *p*-value = 0.0067 and Shannon *p*-value = 0.0053). The depth variable also showed differences for Simpson (*p*-value = 0.005877) and Shannon (*p*-value = 0.02826) indexes, with 200 m having higher indices values than at 2 m (*p*-value for Simpson index: 0.0020 and Shannon index: 0.0114) (Supplementary Table 1).

**FIGURE 4:**
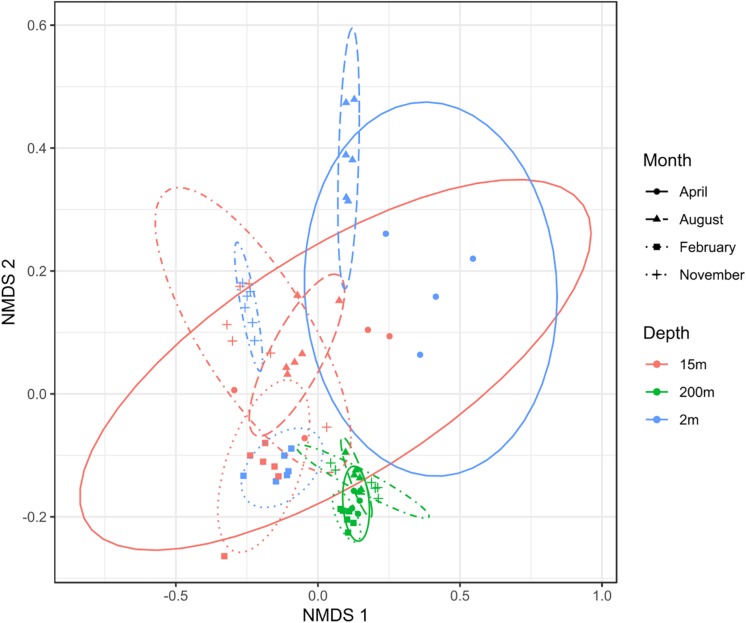
NMDS plot (stress < 0.2) for all sites showing that depth and month variables are responsible for the community dissimilarities.

**FIGURE 5:**
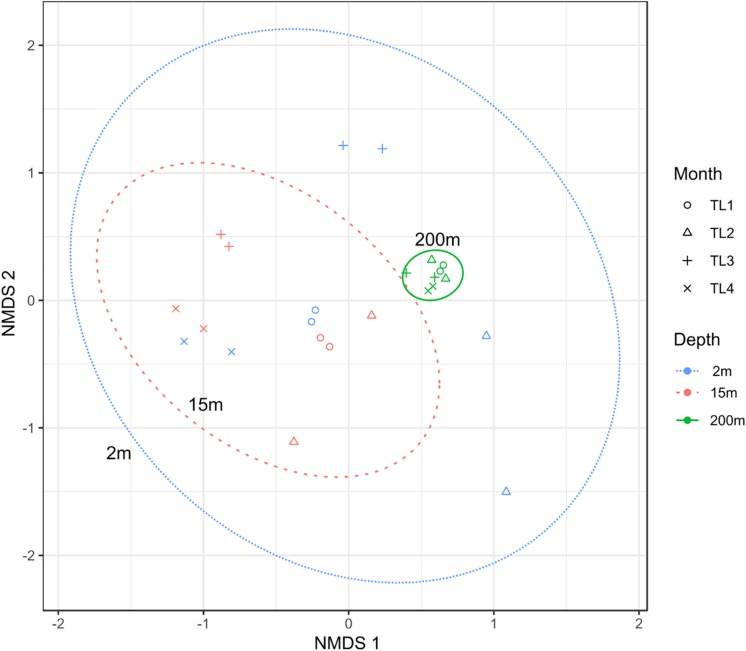
NMDS plot (stress < 0.2) for pt4-SHL2 site showing that depth and month variables are responsible for the community dissimilarities.

**FIGURE 6:**
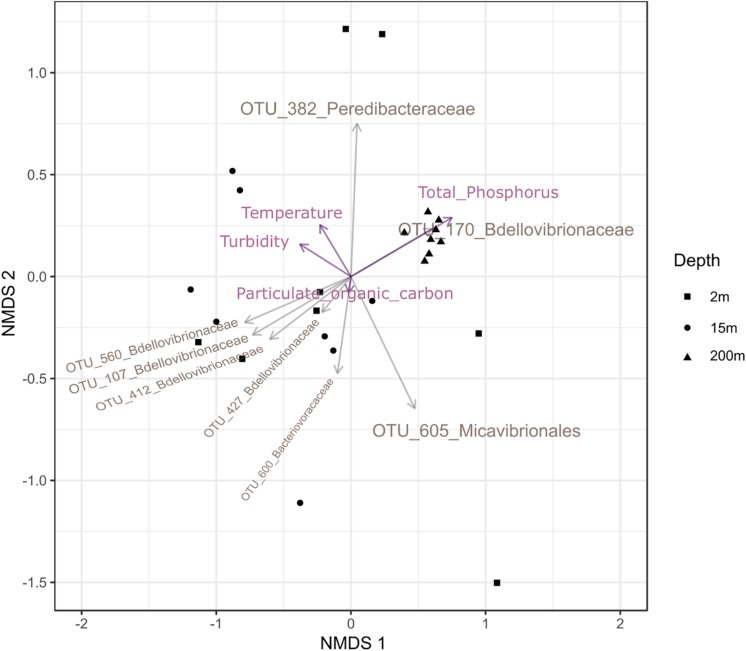
Custom NMDS (stress < 0.2) for pt4-SHL2 site with the abiotic descriptors.

**FIGURE 7:**
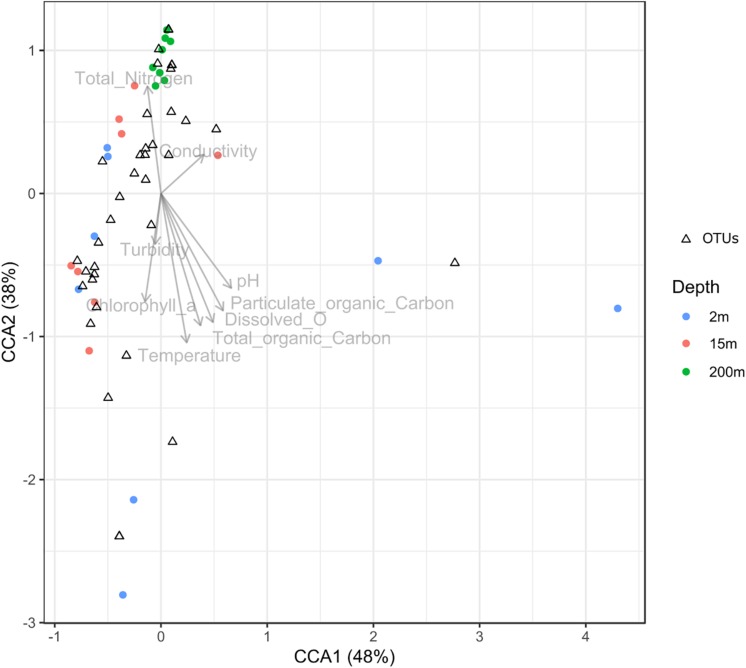
Canonical correspondence analysis (CCA) diagram showing the ordination of OTUs along the first two axes and their correlation with environmental variables.

To test the dissimilarities in the BALOs composition among samples, NMDS, ANOSIM and ADONIS were performed (Figures 4–6). Firstly, all sites were investigated, but then, only pt4-SHL2 was specifically examined with a regular NMDS (*k* = 2) since physicochemical variables were only available for this site which corresponds to the reference station of the lake where the monitoring ecological survey is conducted. A customized NMDS (*k* = 2) was also performed for pt4-SHL2 (Supplementary Tables 2,3). We observed that depth was a key factor affecting BALOs composition as well as the season. When focusing on pt4-SHL2 where we measured a variety of environmental variables, we highlighted that temperature, turbidity, particulate organic carbon and total phosphorus were important to explain BALOs presence, especially the total phosphorus (for the OTU 170 at 200 m for the *Bdellovibrionaceae)*. Then, the canonical correspondence analysis (CCA), performed to infer links between BALO’s OTUs and environmental variables, extracted a high percentage of variance, 48% for canonical axis 1 and 38% for canonical axis 2 (Figure 7). Correlations between the OTUs and the environmental variables were relatively weak. However, some OTUs found at 200 m (i.e. OTU 769, 2465 of the *Bacteriovoracaceae*, OTUs 271, 16036, 21353 of the *Bdellovibrionaceae*, OTU 5245 of the *Peredibacteraceae* and OTU 4836 of the *Micavibrionales*) were associated with total nitrogen, while others were correlated to a lower extent to conductivity, turbidity and chlorophyll-a concentration.

Weight varied between OTUs to explain their relative importance to discriminate seasons or depths. For season, OTU 605 in *Micavibrionales* was more abundant in February than in June and contributed to 23% of the observed community dissimilarity (Supplementary Table 4). The same finding existed between June and August (22%). OTU 382 in *Peredibacteraceae*, more abundant in August, was the most influential to separate February to August and August to November, with an average dissimilarity of 77 and at 81%, respectively. OTU 227 in *Bdellovibrionaceae* contributed to 19% of the dissimilarity between February (where it was the most abundant) and November community composition and the average dissimilarity between these 2 months was 79%. Regarding depth, OTU 382 was also the one which contributed the most to the dissimilarity in the community composition between 2 and 15 m, and between 2 and 200 m. However, there was no significant difference (*p* = 0.8855, Kruskal–Wallis test). In contrast, the difference was significant when considering OTU 112 of the *Bdellovibrionaceae* responsible for the disparity between 15 and 200 m. Its contribution was about 12%, and it was more abundant at 15 than at 200 m. The average dissimilarity between these depths was 83%.

### Relationships Between BALOs and Other Bacteria

**FIGURE 8:**
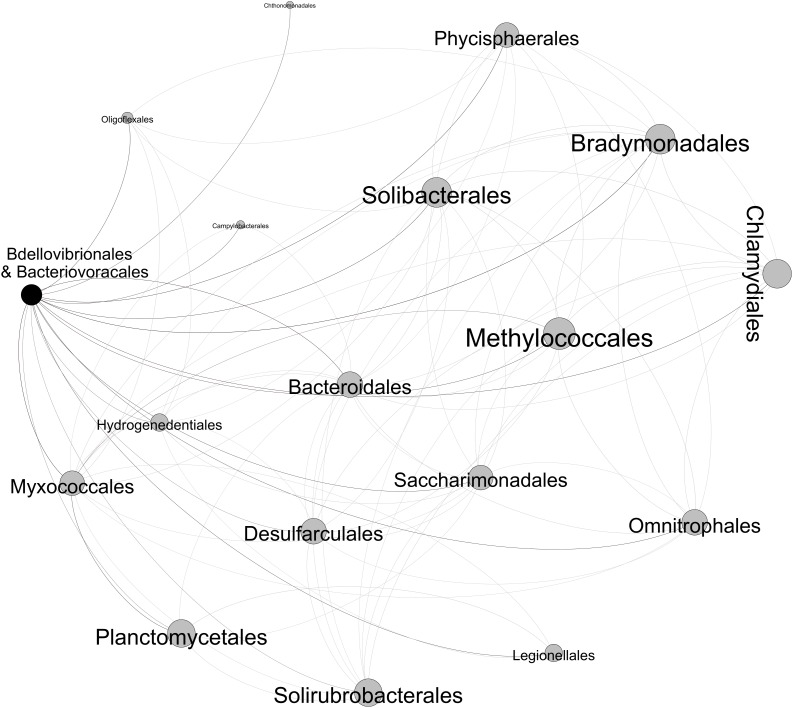
Co-occurrence network revealing the positive relationships between the BALOs and the other bacterial orders.

We focused only on strong (Spearman’s ρ > 0.6) and significant (*p*-value < 0.01) positive correlations between the different bacterial groups (Figure 8). It is noteworthy that *Micavibrionales* did not pass this cutoff. The checkerboard score (C-score) test that was performed to measure the overall co-occurrence in the bacterial OTU table confirmed that the co-occurrence network was non-random. Indeed, the observed C-score (21.103) was higher than the mean value (C-score_mean_ = 20.538, *p*-value < 0.001) expected under the null model. There were 17 positive undirected connections between BALOs and other bacteria, the size of each node being proportional to the number of links (Figure 8). These bacteria were from a variety of orders: *Oligoflexales, Chthonomonadales, Campylobacterales, Phycisphaerales, Solibacterales, Bradymonadales, Methylococcales, Chlamydiales, Bacteroidales, Hydrogenedentiales, Myxococcales, Desulfarculales, Saccharimonadales, Omnitrophales, Planctomycetales, Solirubrobacterales*, and *Legionellales*. The majority of these bacteria are Gram-negative except the *Solirubrobacterales*, which is a Gram-positive. On the other hand, no pieces of information dealing with Gram aspects were found for *Hydrogenedentiales, Saccharimonadales*, and *Omnitrophales*. It is worth mentioning that BALOs were positively correlated with the *Myxococcales*, which are known to include facultative predators.

## Discussion

Our study focused on a specific bacterivorous bacterial group while we are aware that a large variety of predatory bacteria exists and are all likely to play different but significant roles in aquatic ecosystems. The advantage of studying BALOs is that they are represented by a relatively few “species,” are the only group of obligate predators, and they have been well-defined since the early 2000s (Baer et al., 2000, 2004; Davidov and Jurkevitch, 2004; Koval et al., 2015; Hahn et al., 2017). It remains however that, although BALOs are clearly more described and studied (with > 500 publications according to PubMed, October 2019), the importance of their role in natural ecosystems is still unclear.

Using 16S rRNA universal primers targeting bacteria, we focused on the different bacterial predators of bacteria in Lake Geneva. We found that the highest number of OTUs was assigned to *Myxococcales*, followed by BALOs and *Saprospiraceae*. BALOs also included the largest number of reads among all predatory bacteria and seemed to be a relatively homogenous group since the alpha diversity was not significantly different from one site to another. BALOs’ diversity did not change dramatically over-time, while their diversity (Shannon and Simpson indexes) were higher in August than in the other months. Temperature could be a key factor explaining such difference since it is known that BALOs have a limited growth and predation activity at low temperature (<10°C) in the water column and sediment (Williams, 1988; Sutton and Besant, 1994; Kandel et al., 2014; Williams et al., 2016). However, temperature was unlikely responsible for the difference in BALOs’ diversity between summer and fall since it was relatively constant across the two periods. Rather, the difference could be explained by phytoplankton structure and concentrations, since they provide various nutritive substrates for heterotrophic bacteria, some of them being possible prey for BALOs. A higher phytoplankton biomass recorded in November was indeed associated to higher bacterial concentrations at that time (Rimet, 2015; Jacquet unpublished), and likely to changes in the bacterial community composition (Berdjeb et al., 2011), then explaining the increase in BALOs diversity.

While more than half of the sequences obtained for Lake Geneva could be associated to known clusters (Benson et al., 2013) some BALOs were new and constituted new independent clusters. It is noteworthy however that these last OTUs were “out” of the clustering algorithm of Swarm used with a clustering threshold (d) of 13, which is the maximum value of differences allowed between two sequences (Mahé et al., 2015). The median length of BALOs sequence was 376 bp, corresponding to 96.6% of similarity. We are aware that the definition of a “species” is subject to many discussions, and recently, Edgar (2018) suggested to update the identity threshold from 97% (Schloss and Handelsman, 2005) to 99% for full length sequences and to 100% for the V4 hypervariable region of the 16S ribosomal RNA. Therefore, we cannot exclude that our threshold is the best to define *sensu stricto* our BALOs sequences as species. By the way, we noticed that *Bacteriovoracaceae* and *Peredibacteraceae* OTUs clustered together, which might be due to short sequence length and to the identity threshold used.

*Bdellovibrio* and like organisms diversity was higher at depth than surface suggesting these predators occupy a variety of ecological niches (Paix et al., 2019) including cold and dark waters, as phages, maybe because they correspond to sedimenting particles or because they can live on sedimenting material or preys present there. Environmental factors such as temperature, turbidity, particulate organic carbon and merely total phosphorus explained the variation of certain BALOs OTU at 200 m while for others, it was total nitrogen, conductivity, turbidity, and Chlorophyll *a*. Such a disparity among BALOs was also recorded within the seasons. That being said, it is unclear whether environmental variables or prey may better explain BALOs diversity. Since BALOs are obligate predators, we assume that BALOs are more limited by preys than by environmental variables. Indeed, BALOs have been discovered everywhere even in extreme environment (Jurkevitch and Davidov, 2006) such as deep-sea sediment (Williams et al., 2018) and according to Kandel et al. (2014), apart from temperature and salinity, environmental parameters do not offer more insight in explaining BALOs structure. It is also noteworthy to mention here that bacteria diversity was higher at 200 m than at 2 and 50 m (data not shown), likely to offer a higher panoply of potential preys.

As BALOs have different predatory spectrum, some cells being generalists, others specialists, and again others versatilist (Chen et al., 2011), this could explain why we observed the dominance of *Bdellovibrionaceae* in winter in surface, *Micavibrionales* at 15 m and *Bacteriovoracaceae* at 200 m. Comparatively *Micavibrionales* were dominant in summer at 2 and at 15 m along with the *Bacteriovoracaceae* whereas it was *Bdellovibrionaceae* and *Peredibacteraceae* at 200 m. *Peredibacteraceae* were dominant in August whatever the depth and finally November was associated to *Bdellovibrionaceae* at 2 m along with *Bacteriovoracaceae* at 15 and 200 m with *Micavibrionales*. Such dynamics were reported elsewhere (Kandel et al., 2014) and the dominance in summer of the *Peredibacteraceae* was also consistent with our previous study revealing the dominance and high abundance of this group in summer (Paix et al., 2019).

Aside from being the most abundant in terms of reads and OTUs among other predators, BALOs (except for *Micavibrionales*) and *Myxococcales* co-occurred and were positively correlated. No other predators showed similar correlations with BALOs. The rest of the network was mainly composed by Gram-negative bacteria that all may be potential prey for BALOs and *Myxococcales* or simply groups co-varying in response to abiotic factors. This last hypothesis is supported by a recent work where we have isolated new *Bdellovibrio* strains from Lake Geneva that were only able to grow on *Pseudomonas*-like preys (Ezzedine et al., 2020). It is clear however that further work is now required to investigate predator-prey relationships. Kandel et al. (2014) reported that BALOs along with *Myxococcales* were highly abundant in aquaculture zero-discharge systems among other predators. In the same kind of ideas, BALOs were again classified into the Deltaproteobacterial families with the *Myxococcales* (myxobacteria) order a few years ago. In addition, Lowry et al. (2019) reported that *B. bacteriovorus* and *Myxococcus xanthus* shared homologous motility proteins of type IV pili that evolved and diverged to make BALOs a lone predator and *Myxococcus* a social predator. Morgan et al. (2010) described BALOs and myxobacteria as Gram-negative hunter specialists. We then wonder if BALOs and *Myxococcales* are somehow connected to each other or if competition for food has forced them to change their predatory strategy. Myxobacteria can be abundant (Curtis et al., 2007), have a social hunting strategy (wolfpack) *via* swarming motility (Spormann, 1999) and employ chemotaxis-like pathways (Berleman et al., 2008). They attack their prey from distance by producing antibiotics and lytic compounds that kill and decompose it Sudo and Dworkin (1972) and Morgan et al. (2010). In contrast, BALOs are single, obligate and fast swimming predators (Jurkevitch and Davidov, 2006) with a majority being periplasmic (endobiotic) predators. Moreover, chemotaxis was not demonstrated to be used by BALOs, rather, they randomly collide with their prey (Jashnsaz et al., 2017). BALOs’s lytic enzymes are only produced when a prey is invaded (endobiotic) or anchored (epibiotic). Therefore, the impact of BALOs and Myxobacteria in Lake Geneva is likely to be different.

As a conclusion, we believe that despite their low abundance, BALOs can be highly efficient predators (Williams et al., 2016) and could play, at some time and depth, a significant role on structuring the prokaryotic community composition in Lake Geneva. Further studies will have to support this hypothesis.

## Data Availability Statement

*Bdellovibrio* and like organisms generated sequences used to construct the phylogenetic tree can be found under NCBI accession numbers: MN617094, MN617095, MN617096, MN617097, MN617098, MN617099, MN617100, MN617101, MN617102, MN617103, MN617104, MN617105, MN617106, MN617107, MN617108, MN617109, MN617110, MN617111, MN617112, MN617113, MN617114, MN617115, MN617116, MN617117, MN617118, MN617119, MN617120, MN617121, MN617122, MN617123, MN617124, MN617125, MN617126, MN617127, MN617128, MN617129, MN617130, MN617131, MN617132, MN617133, MN617134, MN617135, MN617136, MN617137, MN617138, MN617139, MN617140, MN617141, MN617142, MN617143, MN617144, MN617145, MN617146, and MN617147.

## Author Contributions

SJ designed the study and collected the samples. LJ performed the laboratory work. JE performed sequencing data analyses and statistical analyses. JE and SJ analyzed and interpreted the results. JE and SJ wrote the manuscript with revisions by YD.

## Conflict of Interest

The authors declare that the research was conducted in the absence of any commercial or financial relationships that could be construed as a potential conflict of interest.
