# Supplementary material for: Bdellovibrio and Like Organisms in Lake Geneva: An Unseen Elephant in the Room?
Source: Front Microbiol. 2020 Feb 14;11:98. doi: 10.3389/fmicb.2020.00098 (PMC7034301; doi:10.3389/fmicb.2020.00098)
Supplement: Supplementary file 1 [file Data_Sheet_1.docx]

**
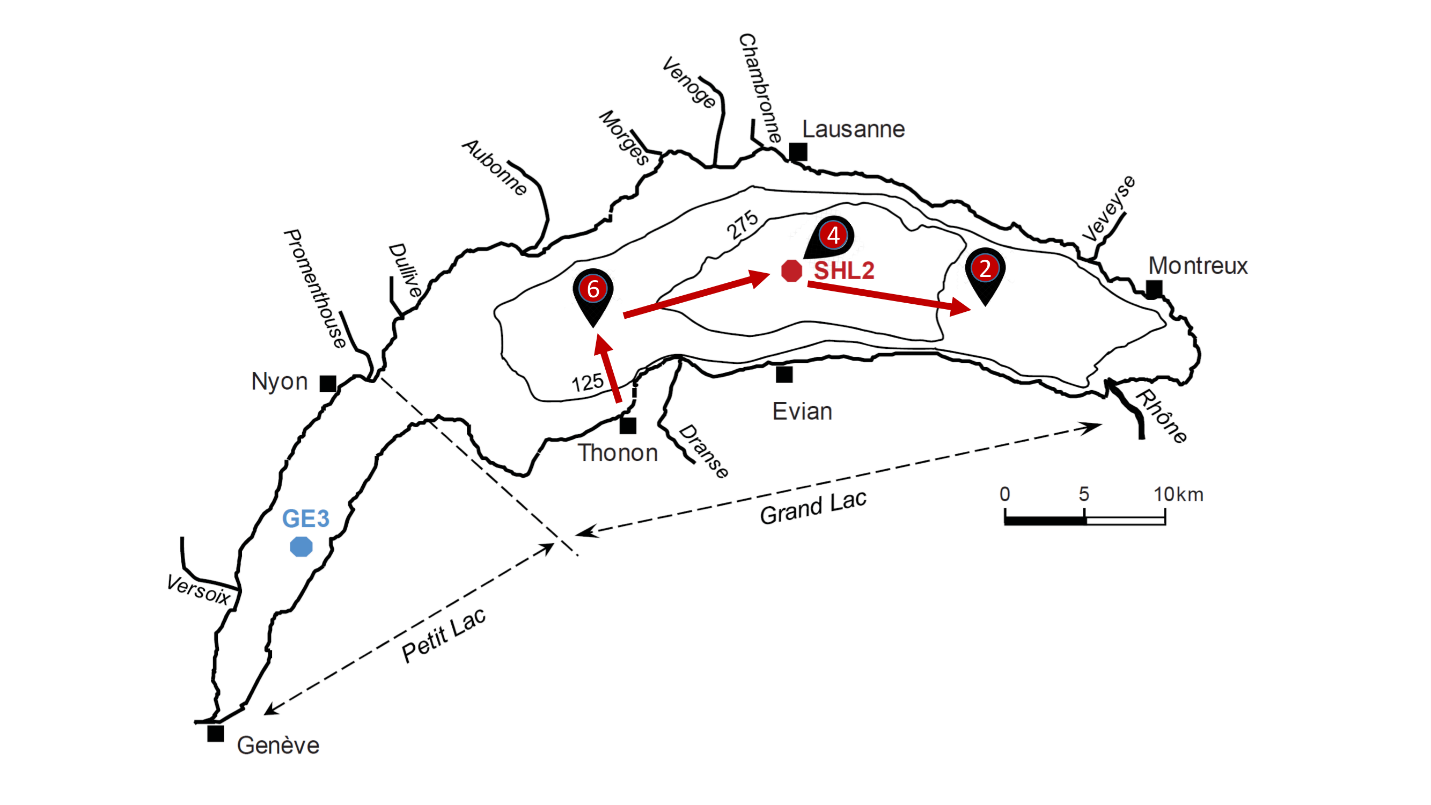
**

**Supplementary Figure 1** Map of Lake Geneva and coordinates of the different sampling points selected during TRANSLEM: Site 2 (N 46° 26.206 / E 006° 46.848), Site 4-SHL2 (N 46° 27.207 / E 006° 35.654) and Site 6 (N 46° 25.061 / E 006° 24.957).

**
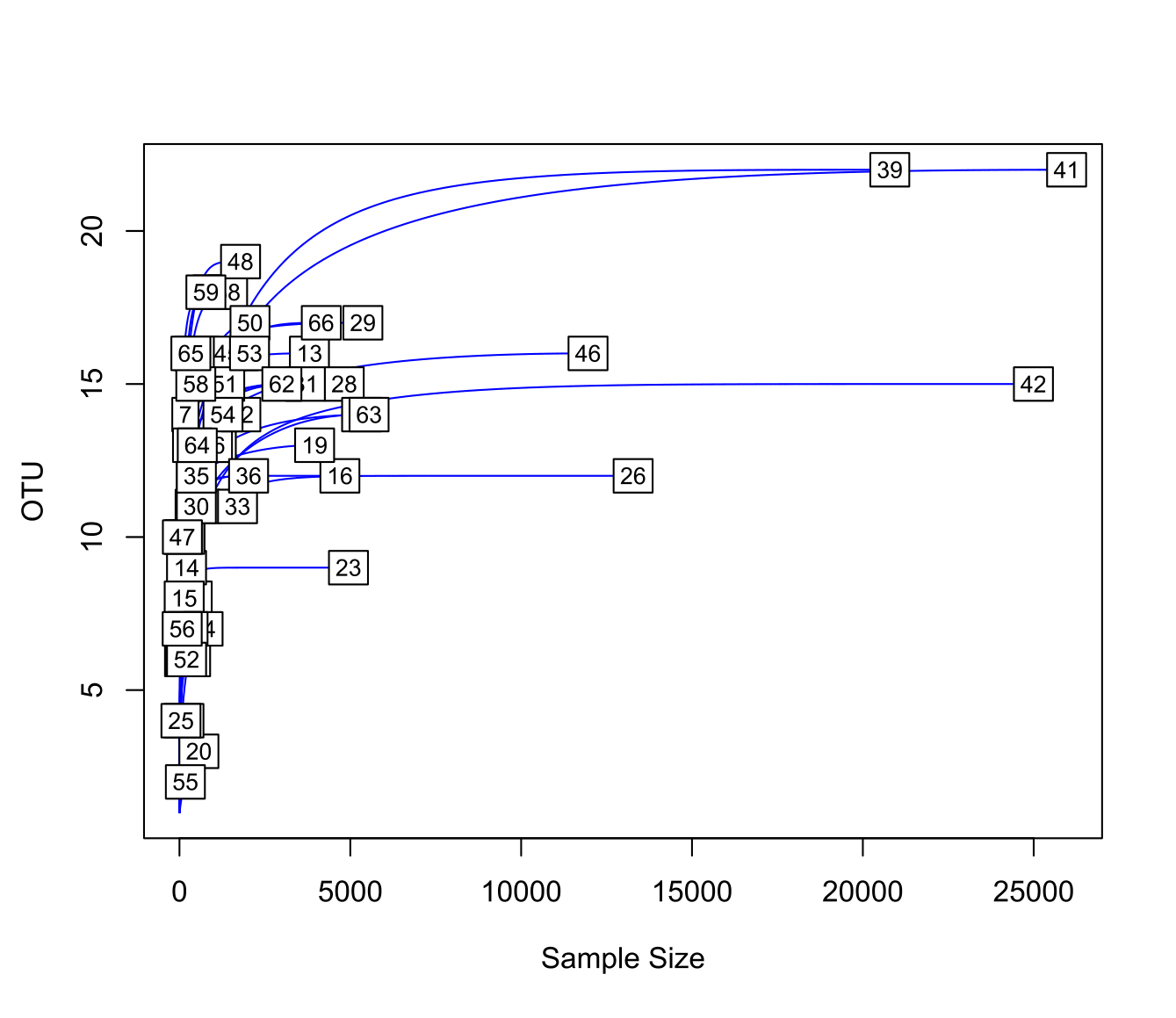
**

**Supplementary Figure 2** Number of BALO reads and OTUs per sample. Sample 41 (November - pt4 - 15m - 5µm+2µm) owns the highest number of reads (25,964) and OTUs (22). Comparatively, Sample 55 (August - pt6 - 2m - 0.2µm) has the lowest number of OTUs, i.e. 2. Sample 25 (June - pt6 - 15m - 5µm+2µm) has the lowest count of reads, i.e. 44. At last, OTU_84_Bdellovibrionaceae was almost everywhere, in 62 out of the 66 samples.

**Supplementary Table 1** Results of the Kruskal-Wallis and Dunn tests performed on alpha diversity indexes in order to detect significant differences between the tested variables (Site, Month, Depth, and Filter). The results show a significant difference for the Month (α= 5%; p-value= 0.009275) and Depth (α= 5%; p-value= 0.0020) variable for Simpson and InvSimpson index. Dunn test indicates that the differences lie within the June and November (α= 5%; p-value= 0.0067) communities for the Month variable, and within 2m and 200m (α= 5%; p-value= 0.002) for the Depth variable. Similar results were obtained for Shannon index. In addition, a significant difference was detected for the filter variable (α= 5%; p-value= 0.03489).

| α diversity index | Condition tested | Kruskal-Wallis test (p-value) | Dunn test  (p-value) |  |
| --- | --- | --- | --- | --- |
| Simpson and InvSimpson | Site | 0.1768 |  | |
|  | Month | 0.009275 * | 0.0067 * | June ≠ November |
|  | Depth | 0.005877 * | 0.0020 * | 2 m ≠ 200 m |
|  | Filter | 0.1192 |  | |
|  | | | | |
| Shannon | Site | 0.1346 |  |  |
|  | Month | 0.01315 | 0.0053 * | June ≠ November |
|  | Depth | 0.02826 | 0.0114 * | 2 m ≠ 200 m |
|  | Filter | 0.03489 | 0.0174 * | 0.2µm ≠ 2µ+5µm |
|  | | | | |
| Pielou | Site | 0.6982 |  | |
|  | Month | 0.7756 |  | |
|  | Depth | 0.1037 |  | |
|  | Filter | 0.08455 |  | |

**Supplementary Table 2** Results of ANOSIM and ADONIS tests. The tests were performed for the three sampled sites (pt2, pt4-SHL2 and pt6) along with the Month, Depth and Filter variable in order to validate the visual interpretation of the NDMS. The results show a significant difference within the Month (α= 5%; p-value= 0.001) and Depth (α= 5%; p-value= 0.001) for all samples sites.

|  | Site | Month | Depth | Filter |
| --- | --- | --- | --- | --- |
| Anosim (p-value) | 0.947 | 0.001 ** | 0.001 ** | 0.423 |
| Adonis (p-value) | 0.891 | 0.001 ** | 0.001 ** | 0.543 |

**Supplementary Table 3** ANOSIM and ADONIS tests performed for the pt4-SHL2 site only, along with the Month, Depth and Filter variable in order to validate the visual interpretation of the NDMS. The results show a significant difference within the Month (α= 5%; p-value= 0.001) and Depth (α= 5%; p-value= 0.001) variables.

|  | Month | Depth |
| --- | --- | --- |
| Anosim (p-value) | 0.001 ** | 0.001 ** |
| Adonis (p-value) | 0.001 ** | 0.001 ** |

**Supplementary Table 4** Results of the Simper test

| Group | | Most influential OTU | Kruskal-Wallis (p-value) | Simper cumulative contributions | Average abundances in each compared treatment | Average dissimilarity between the two treatment |
| --- | --- | --- | --- | --- | --- | --- |
| Month | February – June | 605_Micavibrionales | 1.508e-07 *** | 23% | February < June | 80% |
|  | February – August | 382_Peredibacteraceae | 6.661e-06 *** | 21% | February < August | 77% |
|  | February - November | 227_Bdellovibrionaceae | 7.568e-06 *** | 19% | February > November | 79% |
|  | June - August | 605_Micavibrionales | 1.508e-07 *** | 22% | June > August | 80% |
|  | June - November | 605_Micavibrionales | 1.508e-07 *** | 22% | June > November | 83% |
|  | August - November | 382_Peredibacteraceae | 6.661e-06 *** | 20% | August > November | 81% |
| Site | pt2 - pt4 | 227_Bdellovibrionaceae | 0.9722 | 11% | pt2 < pt4 | 73% |
|  | pt2 – pt6 | 382_Peredibacteraceae | 0.3048 | 13% | pt2 < pt6 | 74% |
|  | pt4 – pt6 | 382_Peredibacteraceae | 0.3048 | 11% | pt4 < pt6 | 77% |
| Depth | 2m – 15m | 382_Peredibacteraceae | 0.08855 | 17% | 2m > 15m | 77% |
|  | 2m – 200m | 382_Peredibacteraceae | 0.08855 | 14% | 2m > 200m | 88% |
|  | 15m – 200m | 112_Bdellovibrionaceae | 3.68e-09 | 12% | 15m > 200m | 83% |
| Filter | 0.2µm - 2µ+5µm | 382_Peredibacteraceae | 0.8934 | 11% | 0.2µm > 2µ+5µm | 75% |

**Supplementary Table 5** Accession numbers of the sequence downloaded from NCBI to construct the phylogenetic tree

| Sequence name | Accession number |
| --- | --- |
| *Bdellovibrio bacteriovorus* strain HD 127 | AJ29276.1 |
| *Bdellovibrio sp.* W strain ATCC 27047 | AJ292518.1 |
| *Bdellovibrio bacteriovorus* strain 109J | M61234.1 |
| *Bdellovibrio bacteriovorus* strain HD100 | NR_027553.1 |
| *Bdellovibrio exovorus* strain JSS | EF687743.1 |
| *Bdellovibrio exovorus* strain MPR11 | MH230062.1 |
| *Peredibacter sp*. Uncultured clone K2DN38 | KT308262.1 |
| *Peredibacter sp.* Uncultured clone C114001412 | JX525305.1 |
| *Peredibacter sp.* Uncultured clone BFB660 | KC545751.1 |
| *Peredibacter sp.* Uncultured clone C114001299 | JX525192.1 |
| *Peredibacter starrii* strain A3.12 | NR_024943.1 |
| *Bacteriovorax stolpii* strain Uki2 | NR_115142.1 |
| *Bacteriovorax sp.* EPC3 | AY294222.1 |
| *Bacteriovorax sp.* EPA | AY294220.1 |
| *Bacteriovorax sp.* F2 | AY294218.1 |
| *Micavibrio aeruginosavorus* strain ARL-13 | DQ186612.1 |
| *Micavibrio sp*. EPB | DQ186613.1 |
| *Vampirovibrio chlorellavorus* strain ICPB 3707 | NR_104911.1 |
|  |  |
